# Sociolinguistic factors affecting performance in the Clinical Skills Assessment of the MRCGP: a mixed-methods approach

**DOI:** 10.3399/bjgpopen17X100713

**Published:** 2017-02-15

**Authors:** Kamila Hawthorne, Celia Roberts, Sarah Atkins

**Affiliations:** 1 Associate Dean for Medicine, Faculty of Health and Medical Sciences, University of Surrey, Guildford, UK; 2 Emeritus Professor in Centre for Language, Discourse and Communication, King's College, London, UK; 3 Associate Professor, Centre for Research in Applied Linguistics, University of Nottingham, Nottingham, UK

**Keywords:** simulated consultations, communication skills, assessment, sociolinguistics, general practice

## Abstract

**Background:**

Differential performance in clinical skills assessments is a widespread phenomenon, for which there remain few explanations.

**Aim:**

To better understand the conversational contexts of simulated consultations and how candidates actually behave in these consultations and to determine sociolinguistic factors for high- and low-performing candidates.

**Design & setting:**

Taking the Membership of the Royal College of General Practitioners' (MRCGP) clinical skills assessment (CSA) examination as a model, this research applied sociolinguistic analyses to case videos of 198 consecutive candidates presenting for the CSA examination.

**Method:**

Using a mixed-methods approach, both quantitative and qualitative sociolinguistics methodologies were combined to analyse video consultations, and findings were compared with those from group discussions with MRCGP examiners.

**Results:**

There is more ‘talk’ in simulated consultations than in real life. On macroanalysis, there was little difference between poor- and well-performing candidates. However, microanalysis found subtle differences in structuring consultations, metacommunication, picking up cues, and misunderstandings with and giving explanations to patients. Formulaic talk, contrary to examiners’ perceptions was more common in successful candidates, but it was personalised and sited appropriately in the consultation.

**Conclusion:**

This is an interactionally demanding form of clinical assessment, that requires giving support to candidates and a more analytic approach to the development of interpersonal skills. Sociolinguistic features of consulting to help trainers and candidates prepare for the CSA are identified.

## How this fits in

Psychometric analysis of performance in the MRCGP’s CSA examination has failed to show the reasons for differential performance that has been the subject of much debate. It is part of a wider international picture in both undergraduate and postgraduate examinations. This sociolinguistic study applies a different lens to performance, allowing understanding and observation of detail of talk that would not otherwise be seen. It is of particular use to the training community and to candidates preparing to sit the CSA examination.

## Introduction

Differential performance in the CSA of the licensing MRCGP exams has been noted since the beginning of this assessment in 2007. UK black and minority ethnic graduates are less likely to pass than UK white graduates, and international medical graduates (IMGs) are less likely to pass than UK candidates.^1^ This research mirrors similar findings in many undergraduate and postgraduate examinations (both written articles and clinical tests) in medicine^2^ and other subjects in higher education,^3^ in the UK and other developed countries.

Psychometric analyses of the marks have failed to show reasons for this difference. It does not appear to be due to bias on the part of examiners or role players (RPs).^4–7^ The researchers used a mixed-methods approach, combining both quantitative and qualitative data, and drawing on sociolinguistics methodology to look at the fine-grained detail of candidate performance. The aim of this research was to better understand the conversational contexts of simulated consultations, how the construct of the CSA determines candidate behaviour, and how candidates actually behave in the CSA. The terminology of ‘high’- or ‘low’-performing candidates was used rather than assuming IMG candidates would perform less well than UK graduates. This study looks at some of the sociolinguistic factors that contrast high- and low-performing candidates and outlines key areas where candidates need preparation for the CSA. It suggests learning strategies that will help both performance in the CSA and in day-to-day real-life consultations.

## Method

Videos of 200 consecutive candidates sitting the CSA between February–March and May 2011 were collected for analysis, together with their marks and examiner feedback. From this bank of 2600 individual cases, a purposive sample of 40 video cases were selected, together with their marking schedules and case notes, to transcribe and analyse in detail. High-, mid-, and low-scoring candidates were selected based on both their overall marks as well as declared information on sex, ethnicity, and place of qualification. Equal numbers of male and female candidates were selected. To enable a focus on communicative issues rather than clinical medical reasons for failure, a clinical advisory panel (made up of experienced MRCGP examiners) was established, whose role was to exclude any cases in the selection that had significant medical errors resulting in failure of the case.

Transcripts of four audiorecorded examiner group feedback sessions were also analysed. In these group discussions, examiners were shown selected clips from the dataset and asked for their opinions on what they had seen. 

**Figure 1.**
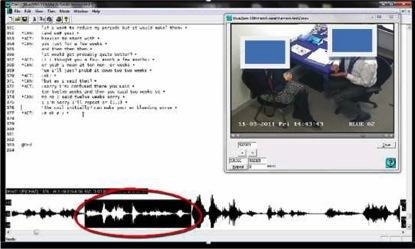
Screen shot of CLAN software used for sociolinguistic analysis.

Video-linked transcription software (CLAN: Computerised Language Analysis) was used to enable both quantitative and qualitative analysis and concurrent close investigation of the words used alongside the linked video and audio file (Figure 1).

The data was subjected to three analytic methods:

1. A broad-based analysis (using the CLAN software), mapping the internal structure of cases, timings of speakers during the case, and the words and phrase frequency in the CSA (known as corpus linguistics [CLs]), comparing these when possible with large reference banks of English used in GP consultations.
2. A microanalysis of the data, looking in detail at interactional alignment, systematically annotating and analysing microinteractional features occurring consistently across cases. For example, how speakers bring the talk into line with one another to achieve a common goal in the consultation, the ways in which ‘formulaic’ talk may arise, and how explanations are structured and conveyed to the RP were also taken into account. The mapping process was based on widely-used sociolinguistic methodology,^8–12 ^with each example identified and agreed by two researchers.
3. Mixed-method and ethnographic analyses of group discussions with MRCGP examiners, and of the case paperwork and marking schedules.

In all of these analyses, patterns of similarity and difference between successful and unsuccessful candidates were compared and analysed.

## Results

Two candidates retrospectively withdrew consent, leaving 198 candidates, (2574 cases in the overall dataset). Table 1 shows their distribution of marks. The 40 cases selected by purposive sampling gave researchers a dataset of just over 85 000 words, sufficient in size to be able to identify patterns of talking. They represented a mix of routine and complex cases, with complex psychosocial or emotionally demanding cases making up just under one-fifth of the dataset.

**Table 1.** Distribution of marks across the 198 candidates consenting to take part in the study

	International medical graduates (EU and overseas)	UK ethnic minority graduates	UK white graduates
Females, *n*	23	29	59
Pass	12	27	59
Fail	11	2	None
Average mark (out of 117)	74.4	87.7	95.7
			
Males, *n*	44	18	25
Pass	14	16	25
Fail	30	2	None
Average mark (out of 117)	67.4	83.6	91.1

The macro and microanalysis of the sociolinguistic data showed that there are particular demands on candidates undertaking simulated consultations, some of which will also help day-to-day consulting quality. Analysis identifies features not widely identified before, and shows that differences between high- and low-performing candidates are not always those that were mentioned by examiners.

The relative decontexualisation of the CSA setting (all cases are ‘new patients’, and the absence of usual practice structures and computers) adds to these requirements, which can be divided into two main themes: how candidates managed the exam format and talk and interaction.

### How candidates managed the exam format

Broad mapping of the ways candidates dealt with the cases shows a striking similarity in general structure and timings across all types of candidate (such as, independent of marks awarded, sex, or ethnicity/country of primary medical qualification), little data gathering takes place after the first 5 minutes. The time constraints require candidates to make rapid inferences from cues given by RPs and these are particularly crucial in the early stages of the consultation. Late data gathering, usually occurring to obtain missed information (even if it resulted in the correct diagnosis and management plan), was predictive of a lower score (occurring in 7 of the 18 poorly-scoring cases) and attracted the feedback of ‘disorganised/unstructured consultation’. Successful candidates structured complex cases more flexibly, often with shorter data gathering as the candidate realised the nature of the case, and allocated more time to explaining or ethical discussion (Table 2).

**Table 2.** Micromoments across the 40 cases

Average number of instances annotated	‘Clear fail’	‘Borderline fail’	‘Borderline pass’	‘Clear pass’
Exam modelling (use of CSA phrases)	6.6	8.5	8.0	9.2
Alignment (expressions of understanding and agreement from the role player)	3.0	3.25	3.0	4.3
Misalignment and misunderstanding (raised by the role player)	5.5	4.0	2.25	2.75

#### Managing role player interruptions

No difference in RP behaviour was discovered between passing and failing case marks. However, RPs interrupted candidates more frequently than in real consultations. Earlier research on real consultations indicates that 33% of interruptions are patient initiated^11^ compared with 50% in the CSA.^13^ The powerful position of RPs in the interaction could be seen in the alternating responses, as they probe and question explanations and clinical plans to get clarification of the candidate’s management (which is part of their role in the exam setting). They were more than twice as likely as candidates to highlight misunderstandings (61 occasions versus 27).

Interruptions in the explanation phase were a sign of poor performance, and were usually the result of inadequate and sometimes chaotic explanations. The combination of poor explaining technique and interrupted dialogue had an additive effect on the overall impression given to a listening examiner: both medically poor and behaviourally uncomfortable. The ‘explaining phase’ was a particularly difficult section of the case, where medical information needs to be tailored to the patient, with high-scoring candidates talking relatively more, and with fewer interruptions than in the rest of the consultation (72–95% of talk time in this phase).

#### Managing the simulation and examiner presence

This research identified a considerable amount of interactional ‘work’ to sustain the talk and illusion of a real consultation and to orient the examiner’s gaze; ‘the heightened mimicry’^14^ which can be rated as a convincing performance while simultaneously being alert to the examiner’s standards. For example, ‘talking about talk’ was a method of signposting both to the ‘patient’ and the listening examiner, where the consultation was heading and what the ‘patient’ was supposed to make of its meaning; for example, ‘tell me what you know about …, then I can build on your knowledge’. This ‘metacommunication’ involves more ‘work’ by the candidate than simply asking open-ended questions, providing a way for the patient’s ‘ideas, concerns and expectations (ICE)’^15^ to be expressed. It was one of many strategies of relationship-building or alignment skills used by successful candidates.^19^

### Talk and interaction

#### The CSA setting

The exam setting affects the amount of talk. Candidates talked more in CSA consultations (68% of the time). Every case was a new patient and there were none of the usual practice structures or computers, than they have in ‘everyday’ GP consultations (50–60% of the time).^16^ The quality of talk was also affected, with candidates needing to make rapid inferences, deal with more questions, and metacommunicate more. CL analysis identified a ‘linguistic fingerprint’ to the CSA; candidates’ use of certain words and phrases indicative of the CSA when contrasted with real consultations. Comparing the dataset with larger reference corpora of general spoken English (Corpus of spoken GP interactions,J Skelton personal communication, 2016 ). showed that some phrases occurred more commonly in CSA consultations than in ‘everyday’ GP consultations.^13^ When case-specific words are filtered out, the remainder are oriented to relationship work in the consultation (for example, ‘OK right OK’/‘bit more about’/‘how can I help’/‘been going on.’^13^

As with the structuring of cases, there was similarity across all candidates in the amount they talked and their overall use of CSA phrases. However, successful candidates showed greater flexibility in the amount they talked in complex cases, which ranged from 45 to 76%. But most of the subtle differences between high- and low-performing candidates were in relationship work and in giving extended explanations.

#### Alignment and misalignment

CL analysis demonstrated that the most frequent CSA phrases were oriented to relationship work in the consultation, the business of creating and maintaining alignment: how to keep the interaction flowing smoothly, and showing some level of explicit affiliation with the RP patient.

Microanalysis of transcripts showed that virtually all candidates experienced moments of misunderstanding and/or misalignment, some measure of behavioural discomfort or confusion. However, overt misunderstandings were rare in the data collected, and there was no evidence that pronunciation caused misunderstandings. Table 2 shows two clear trends: typical CSA phrases were used more with successful candidates than unsuccessful ones and there were more misunderstandings and misalignments among unsuccessful candidates. These were often the result of missing cues which, as well as leading to misalignment, could result in missing the nub of the case.

With about one-third of candidates being IMG, it was pertinent to look for overt cultural misunderstandings that might affect performance in this group. Only one example of possible cultural misalignment was identified in the 40 cases and it involved an IMG candidate explaining genetic inheritance of a condition to a white RP who asked about first-cousin marriage. This example did not cause any overt misunderstanding.

#### Formulaic talk

Examiners commented that some candidates sounded: ‘formulaic’ or ‘clunky’, overusing the common ‘CSA phrases’ identified above. The fact that these CSA phrases were generally seen as formulaic, yet were used more by successful candidates (Table 2), raises the question of *how* they were used. Detailed microanalysis showed that similar phrases were located and delivered differently by high- and low-rated candidates. These phrases appeared formulaic when they appeared as isolated fragments, often with a rapid topic change to some aspect of clinical management. Successful candidates were able to tailor these phrases to the individual conversation and site them correctly in the talk.

Common CSA phrases were also seen as formulaic in the way they were delivered. Differences in how these phrases sounded depended on intonation and other features of speech delivery: pitch, rhythm, and stress, known collectively as ‘prosody’, which can affect shades of meaning in speech. Local English speakers’ use of prosody can differ systematically from that of English speakers from other countries.^8,9^ Local English speakers routinely use what is called ‘an affective contour’ which sounds more natural to a British ear (which is instinctively more familiar with local intonation and voice modulation), while other speakers can sound non-fluent and incomplete, adding to the impression of formulaic consulting, especially if inserted unexpectedly into the conversation.

#### Extended explanations

Successful candidates talked more in the explanation phase (72–95% of the time), and there were fewer misunderstandings and misalignments in this phase. Eighty-one explanation phases were identified from the 40 cases for detailed analysis (Table 3).

**Table 3.** Types of explanation

Types of explanation	*n*		
**Routine clinical explanations**: the majority of cases, nearly all marking the end of a period of data gathering and/or physical examination or discussion of test results	45		
**Demanding clinical explanations:** where an intricately linked description was needed (for example, in explaining a genetic condition), often with several interlinked explanation phases. These were not necessarily complicated by the need to negotiate with the patient, but were difficult through the amount of complex, logically linked information required, as well as taking into account the patient’s response	20		
**Stance saturated:** where the doctor had to balance institutional needs against the patient’s individual need, as part of a joint decision-making process (for example, a patient requesting glucosamine)	10		
**Social or emotional decision-making:** similar dilemmas to ‘stance saturated’ but involving a more personal decision by the patient based on their own presenting issues	6		
Total explanations analysed in the 40 case dataset	81		

Good explanations were logical, linked mini-explanations to causes, and used language smoothly and grammatically to take the patient on an explanatory narrative (Box 1). They also used understandable metaphors from patients’ everyday experiences to illustrate their points; for example, linking vascular problems with plumbing examples.

**Box 1.** What’s in a good explanation?

**Signposting and metacommunication** (for example, indicating what the explanation is going to be about) **Definitions and causality** (using lay terminology and phrases such as ‘which is/means/what it does/it can cause …’) **Narrative structuring** (logical structure to the explanation, as if ‘telling a story’, but also inviting interpretation, or expression of meaning for the patient) **Repetition** (can act as a scaffolding to give a list of symptoms, but also part of the rhythm and the way speech is patterned to make it easier to listen to) **Referential cohesion** (linking one area of the explanation with other areas, with words like ‘it’ or ‘that’s’, but needs to be clear what is being referred to). **Metaphor** (using everyday structures to explain medical terms; for example, a plumbing system to explain vascular problems) **Convergence and dialogue with patient** (constantly checking you and the patient are aligned; for example, ‘what we call’/‘you’re right that’s what I mean/which are the things I was asking you about …’). A way of showing patient centredness even if you are doing most of the talking.

## Discussion

### Summary

This study shows how difficult it is to measure interpersonal skills objectively in the manner of a clinical science. At the macro level, there is little difference between passing and failing candidates. General skills such as how to structure the consultation were mastered by the majority, who all used similar professional language. Successful candidates tended to be better at personalising formulaic phrases commonly heard in the CSA, and making them specific to the case or individual.

The differences became more obvious on microanalysis, where there were a range of communication/interpersonal behaviours, mostly subtle and difficult to analyse by a listening ear in ‘real-time’ examining. They included the ability to inference early on that a case might be complex, and having the flexibility to approach it differently to ensure its requirements are covered adequately, to pick up RP interruptions as cues that either more clarity is needed or that the case is progressing in the wrong direction, and metacommunicating with both the RP patient and examiner concurrently. In addition, poorly performing candidates tended to have more episodes of miscommunication and misunderstandings, and their explanations were less clear. When these small features of interaction occurred cumulatively over the course of a case, they could have important consequences on the marks.

### Strengths and limitations

Sociolinguistic analysis slows down the analysis of CSA cases, allowing the research team to reach understandings and detail of talk that would not otherwise be seen in real-time observation. However, this study only selected cases where there were no serious clinical errors that would have led to failure in the clinical management domain, as its particular focus was in the context of ‘talk’ and interpersonal skills within a clinical consultation, rather than clinical performance. It would be interesting to see if the same issues identified in this study also applied to cases where clinical performance was seen to be poor.

### Comparison with existing literature

The mixed-methods sociolinguistic quantitative and qualitative approach used to evaluate the performance of CSA candidates in the examination was unique, and helps explain some of the reasons for differences in performance that have not been revealed by prior psychometric quantitative analyses.^4–7^ Comparison with other ‘corpus’ of words from objective structured clinical examinations (OSCE) exams for medical students and real life general practice consultations (J Skelton, personal communication, 2016),^10–12^ triangulated the observation that in the CSA, candidates spend relatively more time talking, with explanations in particular being interactionally demanding.

### Implications for preparation for the assessment

Interpersonal skills are a vital component of medical consulting, integral to providing excellent patient-centred care. Developing these skills was a particularly important area requiring candidate support, and the training community needs to be aware of these issues (Box 2). General feedback to ‘improve communication skills’ is not enough, since so many of the features of performance are microfeatures of talk and interaction which arise in the moment of a particular consultation.

A more analytic approach to consulting skills is needed to help trainees notice and work on those features, which were identified as contributing to lower levels of performance. This is an issue for all similar assessments, and not just the CSA. Box 2 summarises some of the sociolinguistic features of consulting that will help trainers and candidates prepare for the CSA and similar clinical examinations. A full report is available online,^13^ and there are detailed free e-learning modules on the RCGP website,^17^ and an RCGP book.^18^

**Box 2.** Take home messages for candidates and trainers

*Planning your learning*
Use videorecordings of your own and others’ consultations to slow down the process so that you can analyse the successful and less successful performance features. Also identify those communicative aspects of the CSA that require a different or heightened performance than that required in real consultations.
*Managing the exam format*
Be aware of the framework of the test in order to navigate the combination of time constraints and clinical performance requirements. Avoid late data gathering. Tune into the likely ‘nub’ or ‘core’ of the consultation by listening carefully and making inferences from (often) subtle RP cues. If you think this case is turning out to have an ethical/legal or negotiating element, be prepared to be flexible with your timings and give more time to the explanation and discussion phases. Be aware that RPs have a more powerful role than real patients and are likely to interrupt more and ask for more clarifications. RP interruptions could be a sign that your explanations are not clear enough. Use ‘metacommunication’, or talking about talk, to show both the RP and examiner how you are structuring the consultation, creating natural speech bridges between sections of the consultation, your intentions in the action you take/talk you use and your attentiveness to interpersonal relations.
*Talk and interaction*
Be aware that the simulated consultation requires more talk from candidates than the average amount of doctor talk in real consultations. Be alert to the amount of relationship work required in the CSA; focus on subtle ways in which alignment can be developed and maintained. Identify misunderstandings and misalignments and reflect on how they could be prevented and repaired. Be aware that much relationship work is done through typical exam phrases, widely perceived as formulaic. All candidates use ‘formulaic’ speech, but to get the most out of it, you need to use it appropriately: at the right point in the interaction and with time for RPs to express their feelings. Customise these phrases so that they sound part of your ‘natural’ conversation, convincing and sincere, rather than an unexpected phrase that has been rote-learned. Focus on the explaining phase of the case, which can be particularly difficult because it requires an extended stretch of talk adapted to the RP’s level of involvement. Explanations require explicit practice, including practising the components outlined in Table 3, using everyday metaphors to take the ‘patient’ on a narrative journey they can understand and take action on.
